# Metallic Spectacles

**Published:** 1866-12

**Authors:** 


METALLIC SPECTACLES.—M. Foucault recently communicated to the French Academy of Sciences the fact that the sun may be viewed through a lens covered with silver leaf. The sun's disc, shorn of its beams, can thus be clearly seen. Subsequently, M. Melscius made a useful application of Foucault's discovery. Having been injured while making an experiment in the laboratory, his eyes were painfully affected by light. In this condition he had recourse to spectacles with black glasses, such as are used by engine-drivers; over these he put green glasses, which answered pretty well; but on further experiment, he found the best method was to use pale-blue goggles, covered with silver or gold film, and these he recommends to all persons troubled with weak eyes.

